# Lobular breast cancer: incidence and genetic and non-genetic risk factors

**DOI:** 10.1186/s13058-015-0546-7

**Published:** 2015-03-13

**Authors:** Laure Dossus, Patrick R Benusiglio

**Affiliations:** 1 Inserm, Centre for research in Epidemiology and Population Health (CESP), U1018, Nutrition, Hormones and Women’s Health team, 94805 Villejuif, France; 2 Université Paris Sud, UMRS 1018, 94805 Villejuif, France; 3 Oncogénétique Clinique, Département de Médecine Oncologique, Gustave Roussy Cancer Campus, 94805 Villejuif, France; 4 Centre Expert National Cancers Rares PREDIR, Hôpital Bicêtre AP-HP, 94275 Le Kremlin-Bicêtre, France

## Abstract

While most invasive breast cancers consist of carcinomas of the ductal type, about 10% are invasive lobular carcinomas. Invasive lobular and ductal carcinomas differ with respect to risk factors. Invasive lobular carcinoma is more strongly associated with exposure to female hormones, and therefore its incidence is more subject to variation. This is illustrated by US figures during the 1987 to 2004 period: after 12 years of increases, breast cancer incidence declined steadily from 1999 to 2004, reflecting among other causes the decreasing use of menopausal hormone therapy, and these variations were stronger for invasive lobular than for invasive ductal carcinoma. Similarly, invasive lobular carcinoma is more strongly associated with early menarche, late menopause and late age at first birth. As for genetic risk factors, four high-penetrance genes are tested in clinical practice when genetic susceptibility to breast cancer is suspected, *BRCA1*, *BRCA2*, *TP53* and *CDH1.* Germline mutations in *BRCA1* and *TP53* are predominantly associated with invasive ductal carcinoma, while *BRCA2* mutations are associated with both ductal and lobular cancers. *CDH1*, the gene coding for the E-cadherin adhesion protein, is of special interest as mutations are associated with invasive lobular carcinoma, but never with ductal carcinoma. It was initially known as the main susceptibility gene for gastric cancer of the diffuse type, but the excess of breast cancers of the lobular type in *CDH1* families led researchers to identify it also as a susceptibility gene for invasive lobular carcinoma. The risk of invasive lobular carcinoma is high in female mutation carriers, as about 50% are expected to develop the disease. Carriers must therefore undergo intensive breast cancer screening, with, for example, yearly magnetic resonance imaging and mammogram starting at age 30 years.

## Introduction

Invasive breast cancer is a heterogeneous disease of two main histological types, invasive ductal carcinoma (IDC) and invasive lobular carcinoma (ILC), IDC being by far the most common. IDC and ILC differ with respect to risk factors, and these differences are often overlooked as researchers and clinicians tend to treat breast cancer as a single, homogeneous entity, or only explore the potential differences between the two types in *post hoc* analyses. In this review, we focus on the specificities of ILC regarding incidence and risk factors. We show that ILC is more strongly associated with exposure to female hormones than IDC and that, as a result, its incidence in the past 25 to 30 years has varied more than that of IDC, depending on environmental and lifestyle factors such as menopausal hormone therapy (MHT). As for genetic risk factors, we show that ILC is often underrepresented in patients carrying mutations in the best-known breast cancer susceptibility genes (*BRCA1*, *TP53*), but that it is the only invasive histological type associated with *CDH1*, the diffuse gastric cancer susceptibility gene.

## Incidence

ILCs represent about 10% of invasive breast cancer cases [1,2]. Patients diagnosed with ILC are, on average, about 3 years older than those with IDC [3]. Compared with patients with IDC, ILCs are generally diagnosed at a more advanced stage, with larger tumor sizes and more frequent lymph node invasion, and are more often estrogen receptor- and progesterone receptor-positive [3]. Studies conducted in the US indicated a 65% increase in the incidence of ILC between 1987 and 1999, while IDC rates increased by only 3% during the same period [1]. However, after 1999, the age-adjusted incidence rates of both ILC and IDC steadily declined [2,4].

More specifically, an average annual decrease of 4.6% for ILC was noted between 1999 and 2004 in 44 American states and the District of Columbia, with the largest drop in 2003 when an 8.5% decrease was observed. The average annual decrease for IDC was smaller, 3.3% for the same five-year period. It is likely that the decreasing use of MHT contributed to this decline, with an acceleration in use reduction from 2002 onwards associated with the publication of the Women’s Health Initiative (WHI) trial results [4,5].

## Environmental and lifestyle risk factors

Most breast cancers are related to female hormones, and therefore any factor that increases exposure to these hormones is a potential risk factor. In particular, reproductive factors associated with increased exposure to endogenous estrogens produced by the ovaries, such as earlier menarche, late menopause, low parity, and late age at first birth, are recognized breast cancer risk factors [6-8]. Similarly, women exposed to exogenous hormones (for example, through MHT or oral contraceptives) are often at increased risk [5,9-12].

Lifestyle factors are also associated with breast cancer. There is an estimated 10% increase in risk per 10 g of ethanol consumed every day [13]. Being overweight or obese is also associated with breast cancer risk, but only in postmenopausal women, with a gain of 5 kg/m^2^ in body mass index (BMI) resulting in an 8% increase in disease risk [13]. On the contrary, excess weight is associated with a decrease in risk in premenopausal women. Again, these associations can be explained by hormonal factors: alcohol consumption and postmenopausal obesity are related to higher circulating estrogen levels [14]. In postmenopause, elevated estrogen levels are most probably due to extraglandular production in the adipose tissue, whereas in premenopause, the decrease in female hormone synthesis associated with anovulatory cycles in obese women likely explains the inverse association with breast cancer [14].

ILC being more often hormone receptor-positive than IDC, one would expect hormone-related risk factors to be more strongly associated with lobular than ductal carcinoma.

## Menopausal hormone therapy

MHT in the form of combined estrogen plus progestin treatment (combined hormone therapy or CHT) most likely increases the risk of breast cancer, whereas the effects of estrogen-only treatments (estrogen hormone therapy or EHT) are less clear [12,15-17]. In 2002 the WHI trial demonstrated that CHT use increased breast cancer risk [5]. In this prospective, randomized primary prevention trial, there was a 26% increase in risk of invasive breast cancer in patients taking CHT. A subsequent, detailed analysis of tumor characteristics concluded that the percentages and distribution of IDC and ILC were similar in the CHT and the placebo group [18]. In the WHI estrogen-alone trial that included women with a prior hysterectomy, use of conjugated equine estrogens was associated with a 20 to 25% decreased risk of invasive breast cancer compared with the placebo group, a reduction in risk that was observed with IDC (−30%) in subgroup analyses, but not with ILC, perhaps because of insufficient statistical power [17,19]. This reduction in risk is consistent with preclinical, cellular and animal models showing that low-dose estradiol can cause tumor regression and apoptosis after prior estrogen deprivation [19-21].

**Figure 1:**
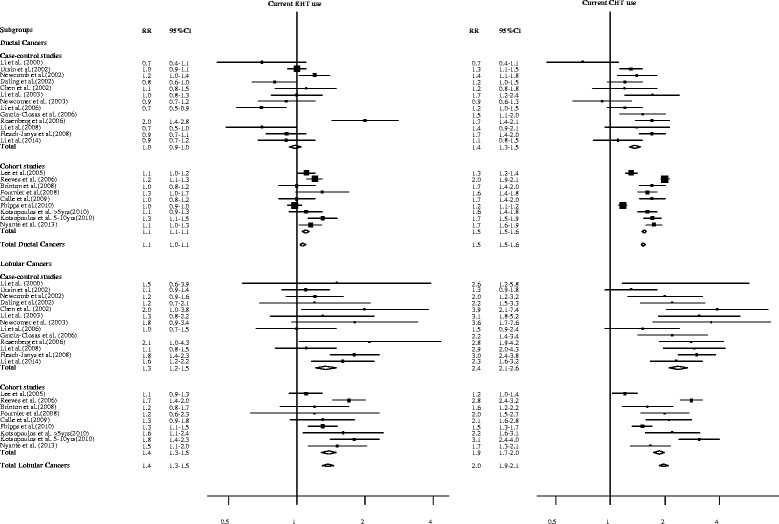
**Relative risks of invasive ductal and lobular breast cancer associated with menopausal hormone therapy in 22 observational studies.** CHT, combined hormone therapy; CI, confidence interval; EHT, estrogen hormone therapy; RR, relative risk.

Twenty-five observational studies (15 case control and 10 cohort studies) have evaluated the association between MHT and breast cancer risk by histological types [22-46]. Although the heterogeneity between histological subgroups was not always formally tested or did not reach statistical significance, a vast majority of these studies showed that MHT was more strongly associated with ILC than IDC. As for the type of MHT, studies that reported associations separately for CHT and EHT are presented in Figure 1. For current CHT use, the relative risk (RR) was generally lower than 2.0 for IDC (overall RR 1.5, 95% confidence interval (CI) 1.5 to 1.6) while it exceeded 2.0 for ILC in a majority of studies (overall RR 2.0, 95% CI 1.9 to 2.1). RR associated with current EHT use varied between 0.7 and 2.0 for IDC (overall RR 1.1, 95% CI 1.0 to 1.1) and between 1.0 and 2.1 for ILC (overall RR 1.4, 95% CI 1.3 to 1.5). Intriguingly, studies that restricted their analyses to estrogen receptor and progesterone receptor tumors still showed a stronger association for ILC than for IDC, suggesting that mechanisms independent of the hormone receptors account for the increased sensitivity of ILC to MHT [36,38,40-42,45,46].

### Oral contraceptives

Oral contraceptives are expected to become less of a risk factor as recent formulations contain less hormones than those available decades ago [12]. To our knowledge, there are no data indicating that oral contraceptives in general have a differential effect on breast cancer depending on the histological subtype.

### Reproductive factors

A recent meta-analysis of individual participant data from 85 studies showed a significantly stronger association with age at menarche for ILC than for IDC (RR per year younger at menarche 1.073 and 1.035, respectively, *P* heterogeneity = 0.0001) [7]. There was also significant heterogeneity between ILC and IDC with respect to the association with age at menopause, with a 3.6% increase in risk of ILC per year older at menopause, versus 2.6% for IDC (*P* heterogeneity = 0.006).

Late age at first birth is associated with an increased risk of breast cancer [8]. Among the 19 studies that explored the association between breast cancer type and age at first birth [32,33,35,42-45,47-58], 14 observed higher risk estimates for ILC than for IDC [33,35,42-45,47,49,51,54-58]. Associations with other reproductive factors, such as parity and breastfeeding, were in general not markedly different between ILC and IDC, although a few studies reported stronger associations with parity for IDC than for ILC [42,43,47,49,51].

### Alcohol

Among eight studies (four case control and four cohort studies) that examined the association between alcohol consumption and breast cancer by histological subtype [33,35,42,45,59-62], two observed a significant heterogeneity between ILC and IDC [33,62]. RRs were higher for ILC than for IDC [33,35,42,45,59,60,62].

### Other risk factors

Four cohort studies [42,43,45,63] and five case–control studies [32,33,35,44,50] examined the associations between anthropometric measures (mostly BMI and height) and risk of breast cancer by histogical type. Overall, none observed significant heterogeneity between IDC and ILC regarding the association with BMI or height. The two studies that examined the association with physical activity did not observe significant heterogeneity between IDC and ILC [45,64].

## Genetic risk factors

Along with environment and lifestyle, a woman’s genetic background contributes to her risk of having breast cancer. Her risk of developing breast cancer increases if she has a family history of the disease. In a re-analysis of 52 studies comparing cancer incidence in relatives of breast cancer cases and in controls, RR for breast cancer increased with increasing numbers of affected first-degree relatives: compared with women who had no affected relative, the RR was 1.80, 2.93, and 3.90, respectively, for women with one, two, and three or more affected first-degree relatives [65]. RR was greatest at young ages and, for women of a given age, was greater the younger the relative was when diagnosed. Twin studies provide evidence that genes contribute substantially to this excess familial risk of breast cancer. Combined data on all twin pairs listed in three North-European registries showed that concordance for breast cancer was two times higher among monozygotic twins, who share all their genes, than among dizygotic twins, who share half their genes [66].

About 90 genes or genetic loci are involved in breast cancer susceptibility in general, through rare, moderate to high penetrance mutations (lifetime risk >20%), the penetrance being the risk for a mutation carrier of developing a disease, or through common variants associated with risks that are only slightly increased compared with the wild-type allele (RR = 1 to 1.5). Mutations and variants are germline; that is, they are part of the genetic identity of the individual. A comprehensive review of genetic susceptibility to breast cancer is beyond the scope of this article, as we are focusing here on the specificities of ILC. Numerous reviews have been written on the issue, and we invite those interested to read two recent and extremely well-written articles [67,68].

Four high-penetrance genes are tested in clinical practice when genetic susceptibility to breast cancer is suspected: *BRCA1*, *BRCA2*, *TP53* and *CDH1*. Germline mutations in *BRCA1* and *TP53* are predominantly associated with IDC, *BRCA2* mutations are associated with both ductal and lobular tumors, while mutations in *CDH1* are exclusively associated with ILC. Mutations in *PTEN* and *STK11* cause, respectively, Cowden and Peutz-Jaeger syndrome, and breast cancer risk is also high in affected females. However, the presenting manifestations are usually not related to breast cancer (for example, macrocephaly, central nervous system abnormalities, mucocutaneous lesions, gastrointestinal hamartomas) and we shall therefore not discuss these two syndromes further [69,70].

### *BRCA1*, *BRCA2* and *TP53*

In the 1990s, linkage analyses and positional cloning in breast and breast-ovarian cancer families led to the identification of *BRCA1* and *BRCA2* [71,72]. Both have a role in maintaining DNA integrity. Mutations confer a high risk of breast and ovarian cancer with estimated breast cancer penetrances of 60% for *BRCA1* and 55% for *BRCA2* by age 70 years [73]. However, not all breast and breast-ovarian cancer families carry a mutation in *BRCA1* or *BRCA2* [74]. For example, 41% of families with four or five cases of breast cancer under the age of 60 years - but no ovarian cancer - are carriers, but that proportion increases to 88% in families with at least four breast cancer cases and one case of ovarian cancer. Mutations are rare in population-based, unselected breast cancer cases. The detection rate was, for example, 2% in a large English series of women diagnosed below age 55 years, although it increased to 12% in the subset of women diagnosed before age 35 years [75]. These are likely underestimates, as the sensitivity of gene analysis techniques was lower in the late 1990s than it is today. Some phenotypic characteristics influence the probability of carrying a mutation in *BRCA1* or *BRCA2*. For example, up to 15% of unselected women with triple-negative breast cancer have a *BRCA1* mutation, while there does not seem to be an association with *BRCA2* [76]. Similarly, the distribution among different breast cancer types varies according to the predisposing gene involved. The CIMBA Consortium analyzed the pathology of invasive breast cancers in 6,893 *BRCA1*/*2* mutation carriers, and found that only 2.2% of tumors associated with *BRCA1* were ILC. In contrast, the proportion of ILCs in *BRCA2* mutation carriers was 8.4%, closer to the characteristics of breast cancers from the general population [1,77].

Li-Fraumeni syndrome is characterized by early onset of a variety of tumors. It is caused by mutations in the tumour-suppressor gene *TP53*. Affected individuals are at increased risk of sarcoma, premenopausal breast cancer, brain cancer, adrenocortical cancer, leukaemia, lymphoma, germ cell tumor, melanoma, lung cancer and cancer of the digestive tract [78]. Cancer risk by age 45 years is about 41% in males and 84% in females; lifetime risk is 73% in males, and approaches 100% in females [79]. The majority of cancers in females are breast cancers, and most breast cancers are diagnosed before age 45 years. Little is known regarding the histological characteristics of breast cancers associated with germline *TP53* mutations, but the two studies that have examined the issue have only shown tumors of the ductal type - and none of the lobular type - out of a total of 48 cancers in mutation carriers [80,81]. These data suggest that *TP53* might predispose exclusively to IDC, and not to ILC. Confirmatory studies are nevertheless needed, and it seems premature at this stage to exclude ILC from the tumor spectrum associated with Li-Fraumeni syndrome.

ILCs are therefore very much underrepresented in carriers of *BRCA1* and *TP53* mutations, while their frequency in *BRCA2* mutation carriers is more similar to that in the general population. This overall underrepresentation of lobular cancers contrasts with observations made in over 40,000 Utah cases with genealogical records showing unusually high levels of familial clustering for ILC, and therefore a higher contribution of genetic, inherited factors compared with IDC [82]. The *CDH1* susceptibility gene likely explains at least some of this excess in familial risk, the remainder being accounted for by genes and loci that are yet to be discovered.

### *CDH1*

*CDH1* is located on chromosome 16q22 and codes for the E-cadherin protein. E-cadherin maintains tissue integrity as it mediates cell-cell adhesion. There is also evidence that forced expression of the protein inhibits the growth of breast cancer cells via mechanisms that are yet to be determined, and that the protein therefore controls cell proliferation in addition to its anti-invasion properties [83,84]. Its tumor-suppression role is limited to breast cancer of the lobular type. Indeed, loss of expression is observed in the majority of lobular breast carcinomas, and, in the few tumors with conserved expression, E-cadherin integrity is impaired [85]. On the contrary, expression is unaffected in ductal breast carcinomas [86]. First-event somatic mutations, with subsequent loss of heterozygosity or promoter methylation, are classically responsible for *CDH1* inactivation following the two-hit loss-of-function model [84]. Therefore, an individual with an inherited, germline mutation in *CDH1* is at increased risk of ILC as a single somatic event is sufficient to generate tumorigenesis.

Readers should be reminded here that *CDH1* was initially known as a susceptibility gene for gastric cancer of the diffuse type, following the identification of germline mutations in Caucasian, Maori and African-American families with multiple affected individuals [87-91]. Like in ILC, E-cadherin inactivation is an early event in diffuse gastric cancer development and, as expected in this context, the histopathological characteristics of diffuse gastric cancer show similarities with ILC, with neoplastic cells permeating the mucosa and wall as scattered individual signet-ring cells or small clusters in an infiltrative growth pattern [87,92,93]. In a collaborative study based on 11 *CDH1* families, the International Gastric Cancer Linkage Consortium showed that the clinical penetrance for diffuse gastric cancer was high, as the estimated risk for carriers of developing the disease was 67 to 83% [94]. Interestingly, that same study observed that, in addition to diffuse gastric cancer, female carriers were also at high risk of ILC [94]. Indeed, there were seven cases of breast cancers in these 11 *CDH1* families, some of them at an early age, and histology, when documented, was systematically of the lobular type. The estimated risk for ILC was 39% by age 80 years. Subsequent studies of families with *CDH1* mutations led to similar conclusions: in four families with a total of 22 breast cancers, all invasive tumors for which a pathological report was available were lobular [95,96]. As for penetrance, a recent estimate derived from 67 mutation-positive families is 56% (P Kaurah and D Huntsman, personal communication).

There is increasing evidence that a personal history of early-onset bilateral ILC or family history of multiple ILC at a young age, in the absence of diffuse gastric cancer in the family, can be associated with *CDH1* germline mutations. Masciari and colleagues [97] described the case of a woman carrier with unilateral ILC at age 42 years, and whose mother had been diagnosed with the same condition at age 28 years. We reported three female cases who presented with bilateral ILC below age 50 years and turned out to carry mutations in *CDH1* [98]. In the only systematic study of women with bilateral lobular breast neoplasia before age 60 years (ILC and/or lobular carcinoma *in situ*), Petridis and colleagues [99] found mutations in 4 out of 50 (8%) women. Schrader and colleagues [100] had previously looked into the issue with discrepant findings, as they only found mutations or potentially causal variants in 4 out of 318 (1%) women with ILC either before age 45 years or regardless of age if there was a family history of breast cancer. However, it was not known how many women actually had a family history of breast cancer of the lobular type, as histology in relatives was not specified. Furthermore, there was no upper age limit for women with familial ILC, and *BRCA1*/*2* mutations had not been excluded in all cases. Cancer geneticists should therefore consider prescribing *CDH1* germline analysis in patients with a personal or family history of multiple pathologically proven early-onset ILC, but no diffuse gastric cancer, as the identification of a mutation would have direct and dramatic clinical implications. The patient would be offered risk-reducing gastrectomy (assuming her ILC has been successfully treated), given the high risk of diffuse gastric cancer [94,101]. Her adult relatives would then undergo targeted genetic analysis to see if they carry the mutation, and those who do would also be offered risk-reducing gastrectomy. Surveillance with upper endoscopy is a poor alternative to prophylactic surgery, except in very specific situations (for example, young athletes wishing to delay surgery for professional reasons, and elderly or frail patients), as this screening modality frequently misses foci of diffuse carcinoma in mutation carriers even when accompanied by multiple random biopsies [102,103]. Large, multicenter studies on the prevalence of *CDH1* mutations in patients and families with multiple cases of ILC are needed.

The high risk of ILC in females carrying a *CDH1* mutation justifies personalized, intensive surveillance. The consensus 2010 *CDH1* paper recommended that breast cancer surveillance be carried out within specific research protocols, and suggested annual magnetic resonance imaging (MRI) and mammogram starting at age 35 years [101]. As ILC risk is close to the overall breast cancer risk seen in carriers of *BRCA1*/*BRCA2* mutations, it seems reasonable to offer the same type of surveillance as a routine procedure, and start screening at age 30 years with annual MRI and mammogram [104,105]. Risk-reducing mastectomy could be an alternative. Updated international recommendations on the management of *CDH1* mutation carriers that will address the issue are expected soon.

### Other genes and future perspectives

Over 80 other breast cancer susceptibility genes and loci have been identified in the past few years, but again none have entered clinical practice either because of the difficulty in interpreting results from sequencing analyses or because the RR associated with the mutated alleles is so low that there is at best limited clinical relevance [67,106]. Only one low-penetrance variant was specifically associated with ILC in a pooled, *post hoc* analysis of 36 case–control studies [106].

## Conclusion

We have reviewed in this article the specificities of ILC regarding disease incidence and environmental, lifestyle and genetic risk factors, and have shown that there were notable differences with IDC. ILC is more strongly related to endogenous and exogenous female hormones and its incidence, therefore, is more subject to variation, depending, for example, on key reproductive factors such as age at menarche or at first pregnancy, or on MHT use. Genetic risk factors vary depending on breast cancer histology, and *CDH1* proves that genes involved in susceptibility to ILC do not have to be involved in IDC susceptibility. The type-specific hypothesis is hardly ever explored in breast cancer epidemiology. It is essential, therefore, that, in the near future, studies start turning their attention specifically to ILC instead of relying on *post hoc* exploratory analyses, or on data extracted from families with mutations predisposing primarily to other cancers. More epidemiological studies are still needed to establish whether associations with other known (physical activity, measures of anthropometry) or still unidentified risk factors differ depending on the histological type. As for genetic studies, large-scale projects focusing on women with unexplained early-onset or familial ILC are urgently needed, as there are certainly many more clinically relevant susceptibility genes to discover. The identification of specific risk factors would help define high-risk groups that could benefit from adapted, personalized screening strategies.

## Note

This article is part of a series on *Lobular breast cancer*, edited by Ulrich Lehmann. Other articles in this series can be found at http://breast-cancer-research.com/series/LBC

## Abbreviations

BMI: Body mass index
CHT: Combined hormone therapy
CI: Confidence interval
EHT: Estrogen hormone therapy
IDC: Invasive ductal carcinoma
ILC: Invasive lobular carcinoma
MHT: Menopausal hormone therapy
MRI: Magnetic resonance imaging
RR: Relative risk
WHI: Women’s Health Initiative
